# Study on the Synthetic Characteristics of Biomass-Derived Isosorbide-Based Poly(arylene ether ketone)s for Sustainable Super Engineering Plastic

**DOI:** 10.3390/molecules24132492

**Published:** 2019-07-08

**Authors:** Seul-A Park, Changgyu Im, Dongyeop X. Oh, Sung Yeon Hwang, Jonggeon Jegal, Ji Hyeon Kim, Young-Wook Chang, Hyeonyeol Jeon, Jeyoung Park

**Affiliations:** 1 Research Center for Bio-based Chemistry, Korea Research Institute of Chemical Technology (KRICT), Ulsan 44429, Korea; 2 Department of Materials Science and Chemical Engineering, Hanyang University, Ansan 15588, Korea; 3 Advanced Materials and Chemical Engineering, University of Science and Technology (UST), Daejeon 34113, Korea

**Keywords:** isosorbide, poly(arylene ether), super engineering plastic, 18-crown-ether, biomass content, thermal dimensional stability

## Abstract

Demand for the development of novel polymers derived from biomass that can replace petroleum resources has been increasing. In this study, biomass-derived isosorbide was used as a monomer in the polymerization of poly(arylene ether ketone)s, and its synthetic characteristics were investigated. As a phase-transfer catalyst, crown ether has increased the weight-average molecular weight of polymers over 100 kg/mol by improving the reaction efficiency of isosorbide and minimizing the effect of moisture. By controlling the experimental parameters such as halogen monomer, polymerization solvent, time, and temperature, the optimal conditions were found to be fluorine-type monomer, dimethyl sulfoxide, 24 h, and 155 °C, respectively. Biomass contents from isosorbide-based polymers were determined by nuclear magnetic resonance and accelerator mass spectroscopy. The synthesized polymer resulted in a high molecular weight that enabled the preparation of transparent polymer films by the solution casting method despite its weak thermal degradation stability compared to aromatic polysulfone. The melt injection molding process was enabled by the addition of plasticizer. The tensile properties were comparable or superior to those of commercial petrochemical specimens of similar molecular weight. Interestingly, the prepared specimens exhibited a significantly lower coefficient of thermal expansion at high temperatures over 150 °C compared to polysulfone.

## 1. Introduction

Poly(arylene ether)s are a type of super engineering plastic (SEP), which is used in various industries owing to its characteristics of high thermal stability (continuous service temperature ≥ 150 °C), low dielectric constant, high chemical resistance, and structural flexibility by ether bonds between the aromatic moieties [1,2,3,4,5,6]. Nucleophilic aromatic substitution (S_N_Ar), a typical method used for the synthesis of arylene ether-type polymers, involves the formation of an ether bond by substituting the leaving group, activated by the *para*-positioned electron withdrawing group, with a nucleophilic monomer, such as phenoxide [7,8]. Commercial polysulfone (PSU) polymers based on such a reaction can be obtained by the polymerization of bisphenol-A (BPA) and aromatic dihalogen monomers in a polar aprotic solvent containing alkali salt, and moisture that disturbs the reaction can be removed by an azeotropic solvent, such as toluene or benzene [9,10].

Because BPA is considered an environmental hormone disruptor, there is some controversy about its negative health effects. The long list of environmental and health issues has motivated the search for sustainable plastics that are partially or entirely derived from biomass feedstocks, replacing petrochemicals. Accordingly, there have been much research and development efforts in the plastics industry to find substitutes for BPA for materials in which BPA is primarily used, such as polycarbonate and epoxy materials [11,12,13]. Isosorbide (ISB) is an anhydrosugar alcohol prepared by a dehydration reaction from sorbitol, which is a hydrogenated sugar that can be obtained by the reduction of biomass glucose, and ISB is the most promising environmentally friendly substitute for BPA [14].

Polymers sourced from biomass have been studied extensively and commercialized as they can replace petrochemical plastic while being environmentally friendly and allowing sustainable growth [15,16]. Various types of polymers have been studied, from the most commonly known polylactic acid made from lactic acid [17,18,19] to aliphatic polymers, such as polybutylene succinate [20,21], and other polymers made from cyclic monomers, such as ISB, 2,5-furandicarboxylic acid, terpene, and lignin [15,22,23,24,25,26,27,28,29,30].

Recently, interest has shifted from commodity polymers to high-value-added engineering plastics, and accordingly, studies on the fabrication of thermally stable polymers that use ISB as the starting material, such as polyester and polycarbonate, have increased [31,32,33,34,35,36,37]. ISB has properties that are more attractive than those of BPA, and in particular, it is known to offer better superior mechanical properties, as well as optical properties. In addition, there have been reports of ISB monomers being used for the synthesis of poly(arylene ether)s, a type of SEP [38,39,40,41,42]. However, the results of such efforts have shown the inability of surpassing a molecular weight (MW) of 10,000 due to the difficulty of moisture control and low reactivity of ISB.

Therefore, there is ongoing research to find commercial, thermally recyclable, and sustainable SEPs, also called high-performance plastics, that utilize bio-derived monomers, e.g., ISB, instead of restricted petrochemicals, e.g., BPA. In the present study, a phase-transfer catalyst was used to polymerize ISB-based poly(arylene ether ketone)s with a high MW without removing moisture from the polymerization constituents. Through an experiment with various controlled parameters, such as the type of halogen monomer, polymerization solvent, time, and temperature, the effect on MW was identified. The biomass contents and thermal degradation stability were compared against commercial PSU. Solution cast transparent films and melt injection molded specimens were prepared to investigate the transparency, mechanical strength, and thermal dimensional stability.

## 2. Materials and Methods

### 2.1. Materials

Among the reagents used in the reaction and analysis, ISB was procured from Roquette Frères (Lestrem, France) and used after recrystallization in acetone, 4,4′-difluorobenzophenone (FBP, 99%) and 4,4′-dichlorobenzophenone (CBP, 99%) were purchased from TCI (Tokyo, Japan) and used after recrystallization in methanol. Potassium carbonate (K_2_CO_3_, 99%) was purchased from Sigma-Aldrich Corp. (St. Louis, MO, USA) and used after grinding into fine particles and drying in a vacuum oven together with phosphorus pentoxide, 18-crown-6 (18C6, 99%), dimethyl sulfoxide (DMSO, 99%), *N*,*N*-dimethlacetamide (DMAc, 99%), 1-methyl-2-pyrrolidinone (NMP, 99%), sulfolane (99%), toluene (99.5%), acetic acid (99%), chloroform (HPLC, 99.9%), and polyethylene glycol (PEG, 400 g/mol) were purchased from Aldrich. Methanol was purchased from Daejung Chemical (Gyeonggi-do, Korea), Commercial PSU was procured from BASF (Ludwigshafen, Germany). All chemicals were used without further purification unless stated.

### 2.2. Polymerization of ISB-Based Poly(arylene ether ketone)s

The polymerization experiment for IK-110 utilizing a phase-transfer catalyst is described as an example. After setting up a mechanical stirrer and Dean–Stark trap on a 100-mL three-neck round-bottom flask, ISB (3.00 g, 20.5 mmol), FBP (4.47 g, 20.5 mmol), K_2_CO_3_ (3.55 g, 25.7 mmol), 18C6 (0.271 g, 1.02 mmol), and DMSO (20.4 mL, 37 wt/v% to the monomer content) were added to the flask. The reactor was set to stir for 24 h at 155 °C under a nitrogen atmosphere to carry out the polymerization process. Upon completion of the polymerization process, DMSO (20 mL) was used to dilute the contents. After cooling to room temperature, the contents were precipitated in a distilled water/methanol mixture (1 L, 50/50 vol%) containing acetic acid (10 mL). To remove any residual salt, the filtered precipitate was redissolved in DMAc solvent, after which it was reprecipitated. The precipitate was washed with distilled water and methanol and then vacuum-oven-dried for 24 h at 80 °C. Product yield (percent per theoretical yield): 6.46 g (96%), *M*_w_: 110,200 g/mol, PDI: 1.68, ^1^H NMR (chloroform-*d*, 300 MHz, ppm): δ 7.83–7.80, δ 7.08–7.01, δ 5.11–5.08, δ 4.97–4.90, δ 4.72–4.70, δ 4.28–4.11.

The polymerization of IK-72 utilizing toluene as an azeotropic solvent and without a phase-transfer catalyst was carried out by the same process as IK-110 described above, except toluene (5 mL) was added to the polymerization medium instead of 18C6. Prior to starting the actual polymerization process, moisture was removed from the reactants by azeotropic distillation at 120 °C for 2 h. Product yield: 6.39 g (95%), *M*_w_: 72,200 g/mol, PDI: 1.73.

Table 1 summarizes the experimental parameters for the polymerization of other polymers.

### 2.3. Characterization

The chemical structures of the polymer and biomass monomer repeating unit-based content by weight were measured using a 300-MHz nuclear magnetic resonance (NMR) spectrometer (Bruker Avance, Billerica, MA, USA). The biocarbon content of the polymer was measured by accelerator mass spectroscopy (AMS, IonPlus, Dietikon, Switzerland). The MW of the polymer was measured by gel permeation chromatography (GPC). The MW relative to standard polystyrene was calculated through an experiment using chloroform as the elution solution at 40 °C in ACQUITY APC XT columns (Waters Corp., Milford, MA, USA). The glass transition temperature of the polymer was measured by differential scanning calorimetry (DSC, Q2000, TA Instruments, New Castle, DE, USA) under a nitrogen atmosphere within the range of 30–250 °C with a temperature variation rate of 10 °C/min. The thermal degradation stability of the polymer was determined by a thermogravimetric analyzer (TGA, Pyris 1, PerkinElmer Inc., Waltham, MA, USA), measuring the sample weight reduction and decomposition temperature by increasing the temperature by 10 °C/min under a nitrogen atmosphere. 

Polymer films were fabricated by casting DMAc solution (10 wt%) in an aluminum dish, followed by drying for two days at room temperature, and then drying for an additional two days in a 100 °C convection oven. The transparency of the polymer film was measured by a UV/vis spectrometer (UV-2600, Shimadzu Corp., Kyoto, Japan). The scratch resistance of the polymer film was determined by a pencil hardness test in accordance with ASTM Standard D3360-00. The films hot-pressed under 100 bar at 200 °C for 5 min were subjected to tensile measurements using a universal testing machine made by Instron (Norwood, MA, USA) with a drawing rate of 10 mm/min. The test specimens were cut into a dumbbell shape, with a length, width, and thickness of 63.50 mm, 3.18 mm, and 100–120 μm, respectively. The fabrication process for the melt injection specimens was as follows. After dissolving the polymer (20 g) and PEG (2 g) in DMAc (200 ml), the mixture was dried for two days in a 100 °C convection oven. After grinding the dried mixture specimen, Haake™ Minijet (Thermo Scientific, Waltham, MA, USA) was used for injection molding into a dumbbell shape under a cylinder temperature of 200 °C, mold temperature of 160 °C, injection pressure of 500 bar, and filling time of 15 s. A tensile strength test on the injection specimens was performed at a drawing rate of 50 mm/min. The coefficient of thermal expansion (CTE) of the polymer film was measured by thermomechanical analysis (TMA, TA Instruments) under a nitrogen atmosphere with a probe force of 20 mN and heating rate of 10 °C/min. The film used for TMA was 15-mm wide, 4-mm long, and 70-μm thick. As a simple experiment for measuring the dimensional stability of the polymer film after exposure to heat, a heat gun (BOSCH, GHG 630 DCE, Gerlingen, Germany) was set-up, and film specimens (1-cm wide, 2.2-cm long, and 100-μm thick) with a dangled 10-g weight were exposed to a temperature of 200 °C from the heat gun at the same distance. After 2 min of heat exposure, changes in outer appearance of the film specimens, and stretch amount from the original length were observed. 

## 3. Results and Discussion

### 3.1. Polymerization Conditions for High MW

**Figure 1:**
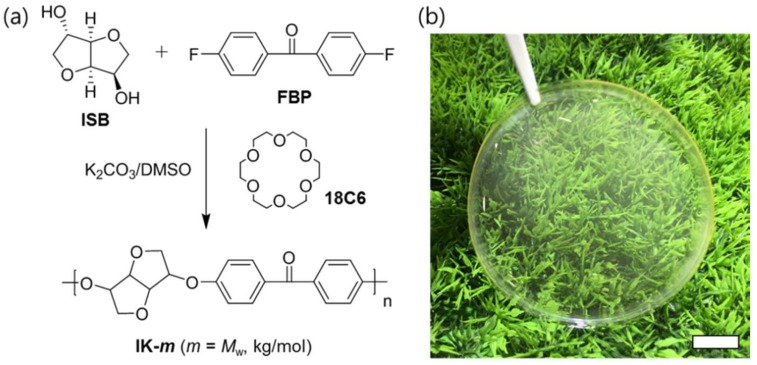
(**a**) Polymerization of ISB-based poly(arylene ether ketone)s of high molecular weight. (**b**) Photograph of IK-110 film (scale bar = 1 cm).

When general S_N_Ar solution condensation polymerization using the toluene-mediated azeotropic distillation technique was applied to the ISB monomers, the number average MW remained at about 10,000 because of the low reactivity of the secondary alcohol and high cyclic oligomer content [38,40]. As a method for overcoming these problems and the hydrophilicity of ISB, our research group attempted to introduce a phase-transfer catalyst to carry out S_N_Ar solution condensation polymerization without the influence of moisture [43]. For the polymerization of ISB-based poly(arylene ether ketone)s with high MW, phase-transfer catalyst 18C6 was added to the reactants to compare its effect (Figure 1a). In addition, the weight average MW (*M*_w_), MW distribution, and glass transition temperature with adjustments to the type of halogen monomer, polymerization solvent, time, and temperature are presented in Table 1.

**Figure 2:**
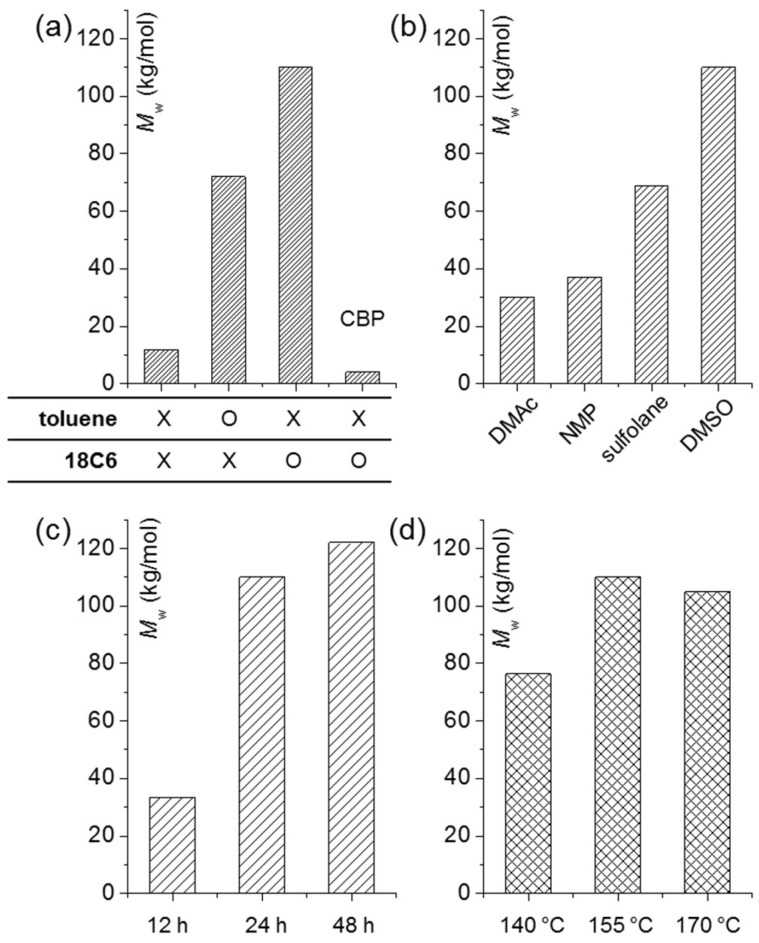
*M*_w_ of polymers depending on various polymerization conditions including (**a**) additives (CBP instead of FBP in the last column), and polymerization (**b**) solvent, (**c**) time, and (**d**) temperature.

When both the phase-transfer catalyst and toluene are absent, *M*_w_ is very low at 12 kg/mol, while under the condition that includes toluene as an azeotropic solvent generally used for BPA-based poly(arylene ether)s, the *M*_w_ of ISB-based polymer reaches 72 kg/mol. Based on the finding that the *M*_w_ of ISB-based poly(arylene ether ketone)s could be increased up to 110 kg/mol by introducing the 18C6 phase-transfer catalyst, it was determined that phase-transfer catalysts play an important role in increasing the MW (Figure 2a). In the polymerization reaction system, the role of 18C6 appears to be the formation of naked anions by capturing potassium ions and the prevention of the hydrolysis of halogen monomers by increasing the efficiency of the substitution reaction. 

Monomers with a fluorine leaving group have a reactivity superior to those with a chlorine leaving group, but it is necessary to consider chlorine monomers from a cost perspective. In the comparative experiment using CBP monomers in place of FBP, CBP and ISB showed a significant decline in reactivity (*M*_w_ of IK-4 = 4.0 kg/mol, Figure 2a). Generally, in the S_N_Ar reaction, the fluorine leaving group has a greater electronegativity, and thus, it is known to play a larger role in lowering the activation energy of the addition reaction by stabilizing the Meisenheimer complex, more so than the chlorine leaving group [44]. The difference in such a role would appear more prominently if ISB with a low reactivity was applied. Among polar aprotic solvents used as a polymerization solvent, selecting the solvent suitable for the polymerization of ISB is also a critical factor. When polymerization was carried out using commonly known solvents, such as DMSO, DMAc, NMP, and sulfolane, the MW appeared in the high-to-low order of DMSO > sulfolane > NMP > DMAc, implying that the highest MW was achieved when DMSO was used as the polymerization solvent (Figure 2b). DMSO solvent also has the advantage of being very cost-competitive. 

Because polymerization time and temperature are factors directly linked to the process operating cost and productivity, the process of finding the optimal conditions is essential. As shown in Figure 2c,d, polymers with a polymerization time of 12 h show a *M*_w_ of only 34 kg/mol, but when the time is increased to 24 h, *M*_w_ shows a large increase to 110 kg/mol. When the time is extended to 48 h, *M*_w_ increases slightly to 122 kg/mol. Accordingly, the optimal polymerization time is confirmed to be 24 h. At a polymerization temperature of 140 °C, *M*_w_ is only 76 kg/mol, but at 155 °C, *M*_w_ increases to 110 kg/mol, while at 170 °C, *M*_w_ actually decreases to 105 kg/mol. The optimal polymerization temperature is confirmed to be 155 °C. It appears that the reverse reaction rate by transesterification is higher at higher temperatures above 155 °C. 

### 3.2. Determination of Biomass Contents of Polymers

**Figure 3:**
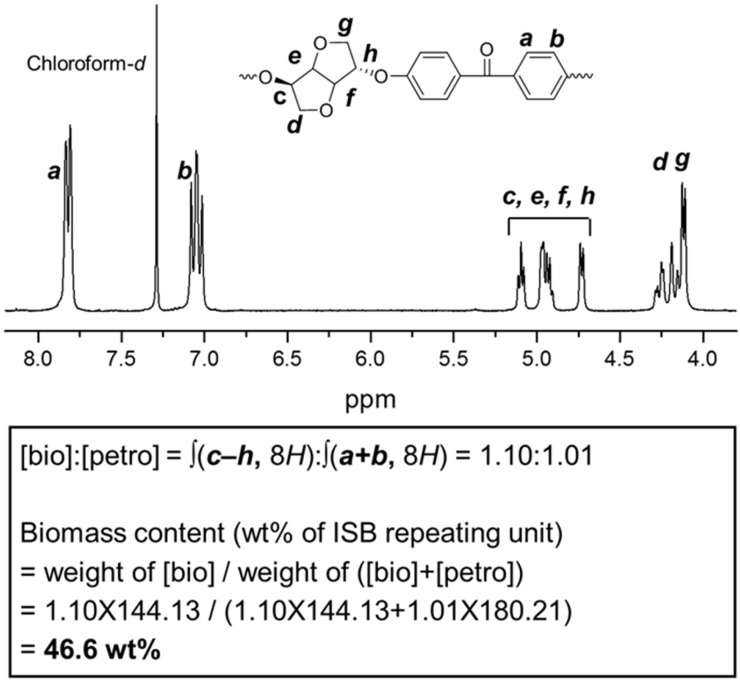
^1^H-NMR spectrum of IK-110 and calculation of biomass content.

The biomass monomer repeating unit-based weight content in the synthesized polymers could be calculated easily by converting the integral ratio of the repeating units of ISB and FBP that appear in the NMR to the weight ratio without detecting the biocarbon content by AMS. In Figure 3, the integral ratio was calculated to be 1.10:1.01, which is close to the theoretical equivalent of ISB and FBP of 1:1. By multiplying the MW of the repeating unit by this, the biomass content representing the percentage of the weight of biomass-based repeating units relative to the total weight was calculated to be 46.6 wt%. This experimental result is very close to the theoretical ISB-based biomass weight content of 44.4%. 

Although the method of using NMR for the calculation of biomass weight content may be simple, it is an indirect method. A more direct method involves using AMS to measure biocarbon content based on radioactive carbon isotope-14 (^14^C) [45,46]. Assuming all ISB introduced into the polymerization process was used to form repeating units in the polymer, the theoretical biocarbon content was calculated as 31.6% using Equation (1) shown below. 

(1) $$\begin{array}{l} \mathrm{Bio}-\mathrm{carbon}\;\mathrm{content}\;\mathrm{from}\;\mathrm{ISB}\;\mathrm{of}\;\mathrm{IK}-110\\ =\frac{\mathrm{Number}\;\mathrm{of}\;C\;\mathrm{from}\;\mathrm{ISB}\;\mathrm{repeating}\;\mathrm{unit}}{\mathrm{Number}\;\mathrm{of}\;C\;\mathrm{from}\;\mathrm{total}\;\mathrm{repeating}\;\mathrm{unit}}\times 100=\frac{6}{6+13}\times 100=31.6 \end{array}$$

The percentage of modern carbon (pMC) content of IK-110 measured in accordance with the ASTM D6866-18 standard was 32.17%, and the biocarbon content calculated with an atmospheric correction factor was 32%, both of which are close to the expected value. In contrast, the biocarbon content in PSU, the petrochemical polymer used as the control, was calculated to be 0% (Table 2).

### 3.3. Thermal Degradation Stability Comparisons of Polymers from ISB with BPA

**Figure 4:**
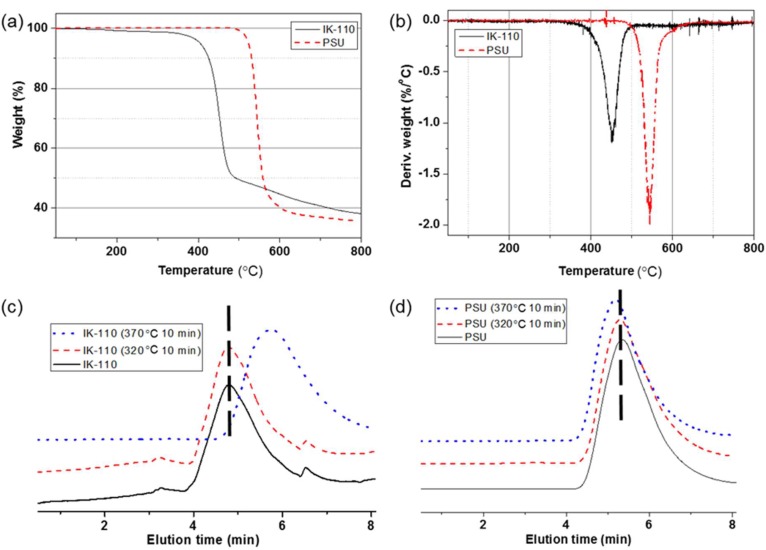
(**a**) Thermogravimetric analyzer (TGA) and (**b**) DTG curves of IK-110 and PSU. Chloroform-GPC profiles of (**c**) IK-110 and (**d**) PSU after thermal stress.

When the thermal stability of IK-110 was compared to the commercial PSU material by TGA, the difference in the 5 wt% weight reduction temperature (*T*_d5_) and maximum decomposition temperature (*T*_max_) were both approximately 100 °C between the materials (Figure 4a,b, Table 3). This is believed to be attributable to the heteroaliphatic ISB-based polymer having a greater decline in thermal stability than aromatic BPA-based polymers, rather than ketone-based polymers having a slightly lower thermal degradation stability than sulfone-based polymers [47]. At 320 °C, which is close to 100 °C below *T*_d5_, the weight reduction rate in IK-110 after 1 h of exposure is 2.5%, but at 370 °C, the weight reduction rate is 50.3%. Considering the weight reduction rate in commercial PSU was only 0.1%, this confirms that a significant amount of thermal decomposition had occurred. With respect to changes in *M*_w_, there is no significant change in *M*_w_ in IK-110 after 10 min of exposure at 320 °C, but after 10 min of exposure at 370 °C, *M*_w_ decreases by approximately 70% (Figure 4c,d). Based on the thermal degradation stability experiment, it was determined that pure ISB-based poly(arylene ether ketone) with no thermal stabilizer has a more inferior thermal stability than aromatic PSU polymers.

### 3.4. Preparation of Solution Casted Films and Melt Injection Molded Specimens for Mechanical Performances 

In previous studies, film fabrication was difficult because of the low MW of ISB-based poly(arylene ether)s. However, in the present study, polymerization with a high MW was possible, which enabled the fabrication of a transparent and strong film by the solution casting method (Figure 1b). As an example, the film fabricated using IK-110 polymer showed a transmittance of 97.2% at 550 nm, which is similar to that of commercial PSU (99.4%). Moreover, when the scratch resistance of the film was tested by a pencil hardness test, the results indicated HB, similar to that of PSU (Table 3).

**Figure 5:**
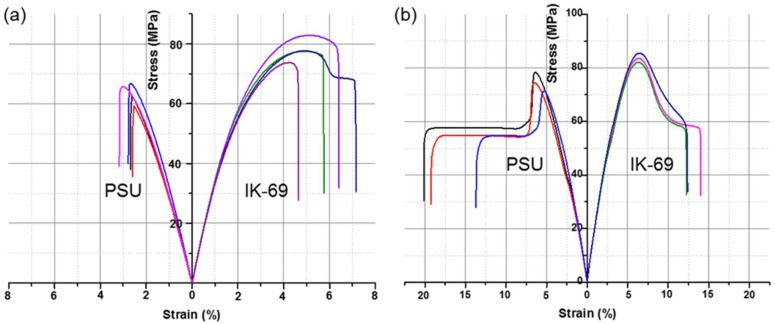
Tensile stress–strain curves of PSU and IK-69 ((**a**) solution cast films, (**b**) melt injection molded specimens).

To compare the tensile properties against the commercial PSU material, solution cast film was fabricated using an IK-69 material with similar MW. The tensile strength test was performed after minimizing defects with additional heat treatment by hot pressing. As shown in Figure 5a and Table 4, under the same film fabrication conditions, the IK-69 film shows a higher Young’s modulus (3.7 GPa), ultimate tensile strength (78 MPa), elongation at break (6.0%), and toughness (3.5 MJ/m^3^) than PSU with respective values of 3.1 GPa, 64 MPa, 2.8%, and 1.1 MJ/m^3^. Moreover, melting processing with synthesized ISB-based polymer is difficult as it does not melt readily even at temperatures above the glass transition temperature. However, the processability improves by adding a plasticizer. The processing temperature was dropped to 200 °C by adding 10 wt% of PEG relative to the polymer, and dumbbell-shaped specimens were prepared by an injection process. When the tensile properties of the prepared specimens were compared, IK-69 showed a higher Young’s modulus (2.2 GPa), ultimate tensile strength (84 MPa), lower elongation at break (13%), and toughness (7.8 MJ/m^3^) than PSU with respective values of 2.0 GPa, 74 MPa, 18%, and 9.3 MJ/m^3^ (Figure 5b). It was determined that superior tensile properties by melt injection were achieved because the specimen had fewer defects than those of film fabrication by solution casting, in addition to a better molecular alignment because of the strong injection pressure and the addition of a plasticizer. 

### 3.5. Thermal Dimensional Stability of Bio-SEP Meriting over Petrochemical PSU

**Figure 6:**
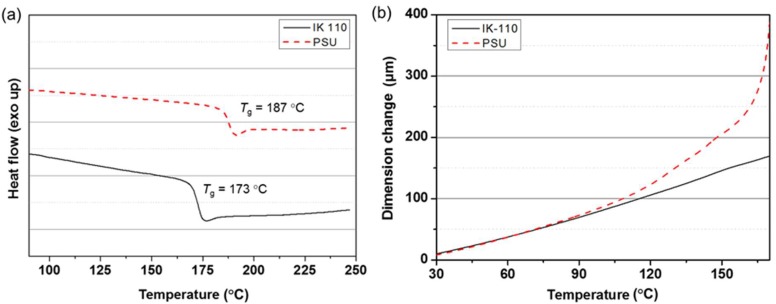
(**a**) DSC and (**b**) CTE curves of IK-110 and PSU films.

The DSC results indicate that despite being a ketone-based poly(arylene ether), i.e. polyether ether ketone (PEEK), a melting point did not appear, because of the copolymerization with ISB (Figure 6a). When changes in the CTE of IK-110 and PSU films were observed by TMA, IK-110 interestingly displayed much more stable thermal extension behavior than PSU, despite having a lower glass transition temperature (*T*_g_ of IK-110 = 173 °C, *T*_g_ of PSU = 187 °C) (Figure 6b). At a low-temperature range of 30–80 °C, the CTE of IK-110 is 53.2 ppm/K, which is approximately 20% lower than 65.4 ppm/K shown by PSU. At a temperature range of 80–170 °C, close to the glass transition temperature, the CTE of IK-110 is 70.4 ppm/K, which is approximately 70% lower than 227 ppm/K shown by PSU. 

The reason the CTE of amorphous SEP containing ISB is lower than that of BPA-based polymers can be attributed to the unique chemical structure of ISB. According to quantum chemical calculations described in previously published articles, a fused aliphatic bicyclic ring exhibits considerably stronger geometrical restraint than the planar benzene structure of BPA, therefore, requiring a greater amount of energy for the same degree of alteration [48,49,50]. The ISB-based polymer with a higher Young’s modulus than the BPA-based polymer can be explained by the same principle.

**Figure 7:**
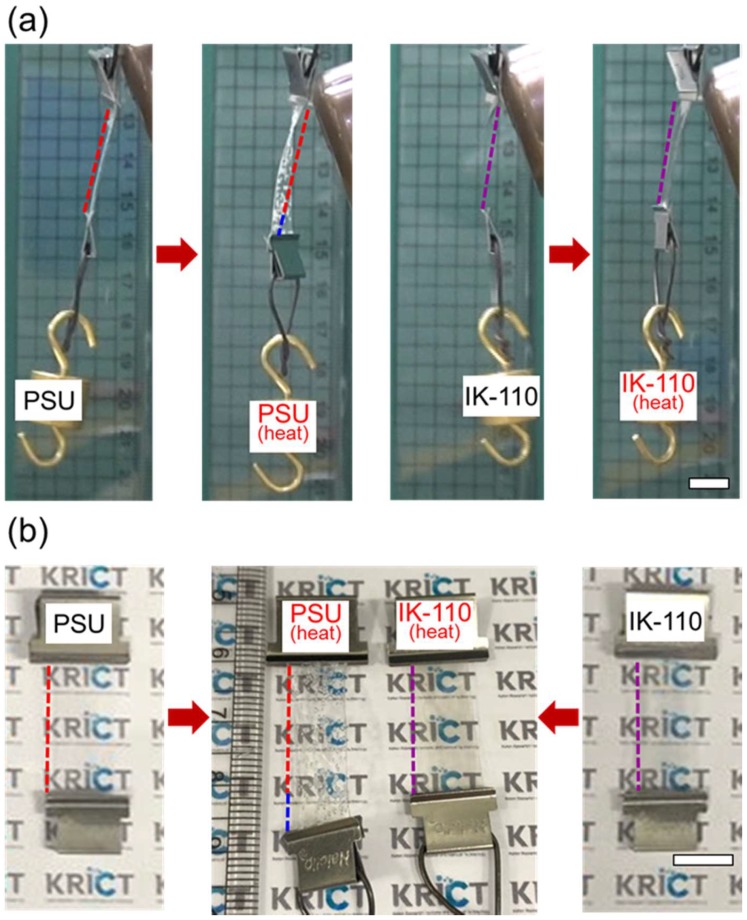
(**a**) Thermal dimensional stability experiment by heat-gun, and (**b**) photographs of PSU and IK-100 films before and after heat exposure to 200 °C for 2 min (scale bar: 1 cm).

As an experiment for visualizing the high dimensional stability of ISB-based SEP films at high temperatures, polymer films with a dangled 10-g weight were exposed for 2 min to a temperature of 200 °C generated by a heat gun to look for variations in the appearance and length of the films (Figure 7a). The PSU film developed bubbles immediately after being exposed to 200 °C hot air, and its length had stretched by 27% within 2 min. In contrast, the IK-110 film maintained its transparency and showed no change in length (Figure 7b).

## 4. Conclusions

In the present study, biomass-derived ISB as a monomer, and crown ether as a phase transfer catalyst was introduced for the synthesis of poly(arylene ether ketone)s with high MW. The optimal polymerization conditions were identified as using fluorine-type monomer as the halogen monomer, DMSO as the polymerization solvent, 155 °C as the polymerization temperature, and 24 h as the polymerization time. Biomass-derived ISB-based polymer showed a ^14^C-based biocarbon content of 32%. The synthesized polymer showed weaker thermal degradation stability than aromatic PSU. However, its high MW enabled the fabrication of a transparent film while exhibiting comparable or better tensile properties and a considerably superior thermal dimensional stability, as compared to PSU. Applications of transparent, highly heat-resistant next-generation materials are expected from such bio-based SEP materials. 

## Figures and Tables

**Table 1:** Polymerization information and results.

Entry (IK-*m*) *^a^*	Halogen *^b^*	18C6 *^c^*	Toluene *^d^*	Solvent *^e^*	Time/Temperature (h/°C) *^f^*	*M*_w_ (kg/mol) *^g^*	PDI *^g^*
IK-12	FBP	X	X	DMSO	24/155	11.6	1.86
IK-72	FBP	X	O	DMSO	24/155	72.2	1.73
IK-110	FBP	O	X	DMSO	24/155	110	1.68
IK-4	CBP	O	X	DMSO	24/155	4.0	1.65
IK-30	FBP	O	X	DMAc	24/155	30.0	2.21
IK-37	FBP	O	X	NMP	24/155	37.0	2.19
IK-69	FBP	O	X	sulfolane	24/155	68.8	1.62
IK-34	FBP	O	X	DMSO	12/155	33.5	2.11
IK-122	FBP	O	X	DMSO	48/155	122	2.08
IK-76	FBP	O	X	DMSO	24/140	76.3	1.56
IK-105	FBP	O	X	DMSO	24/170	105	2.06
PSU	-	-	-	-	-	63.7	1.64

*^a^* Polymers synthesized in this study are designated as IK-*m*, where *m* denotes *M*_w_ in kg/mol. PSU is polysulfone supplied by BASF. *^b^* FBP is 4,4′-difluorobenzophenone and CBP is 4,4′-dichlorobenzophenone. *^c^* 18C6 fed with 5 mol% per ISB. *^d^* Toluene fed for azeotropic distillation of water. *^e^* Amount of solvent fed was 37 wt/v%. *^f^* Polymerization time and temperature. *^g^* Determined by chloroform-GPC using polystyrene standards (refractive index (RI) detector).

**Table 2:** ^14^C-based biomass carbon content measured by AMS.

	pMC *^a^*	Biocarbon Content (%) *^b^*
IK-110	32.17	32 (±3)
PSU	0.24	0 (±3)

*^a^* Percentage of modern carbon. *^b^* Measured twice and corrected by a factor of 100.5 (ASTM D6866-18, method B).

**Table 3:** Thermal stability, transparency, hardness, and thermal expansion of polymers.

	IK-110	PSU
*T*_d5_ (°C) *^a^*	411	522
*T*_max_ (°C) *^b^*	454	545
Weight loss at 320 °C for 1 h (wt%) *^c^*	2.5	0.1
Weight loss at 370 °C for 1 h (wt%) *^c^*	50.3	0.1
*M*_w_ after 10 min at 320 °C (kg/mol) *^d^*	114	63.3
*M*_w_ after 10 min at 370 °C (kg/mol) *^d^*	29.8	63.5
Transmittance at 550 nm (%) *^e^*	97.2	99.4
Pencil hardness	HB	HB
CTE (ppm, 30–80 °C) *^f^*	53.2	65.4
CTE (ppm, 80–170 °C) *^f^*	70.4	227

*^a^* Degradation temperature for 5% weight loss was measured by TGA with a heating rate of 10 °C/min (with N_2_). *^b^* Maximum degradation rate temperature. *^c^* Measured by TGA with N_2_. Temperatures were ramped to the target temperature at a heating rate of 20 °C/min, followed by an isotherm at the target temperature for 1 h. *^d^* Determined by chloroform-GPC using polystyrene standards (RI detector) after holding the samples at the target temperature for 10 min with N_2_. *^e^* Thickness of 70 μm. *^f^* Measured by TMA with a heating rate of 10 °C/min (2nd Scan).

**Table 4:** Tensile properties of PSU and IK-69.

Type	PSU				IK-69			
	Young’s Modulus (GPa)	UTS (MPa)	Elongation at Break (%)	Toughness (MJ/m^3^)	Young’s Modulus (GPa)	UTS (MPa)	Elongation at Break (%)	Toughness (MJ/m^3^)
Solution casted film	3.1 (± 0.09)	64 (±1.7)	2.8 (±0.13)	1.1 (±0.09)	3.7 (± 0.05)	78 (±2.2)	6.0 (±0.73)	3.5 (±0.57)
Injection molded specimen	2.0 (± 0.03)	74 (±2.1)	18 (±2.0)	9.3 (±1.3)	2.2 (± 0.03)	84 (±1.0)	13 (±0.6)	7.8 (±0.34)
